# Editorial: Access and barriers to reproductive health services among immigrants and populations in conflict zones

**DOI:** 10.3389/frph.2025.1674390

**Published:** 2025-12-10

**Authors:** Comfort Z. Olorunsaiye, Negussie Boti Sidamo, Sue Anne Bell

**Affiliations:** 1 Department of Public Health, Arcadia University, Glenside, PA, United States; 2 College of Medicine and Health Sciences, Arba Minch University, Arba Minch, Ethiopia; 3 School of Nursing, University of Michigan, Ann Arbor, MI, United States

**Keywords:** family planning, maternal health, abortion care services, sexual and reproductive health, unintended pregnancies, gender-based violence, contraceptive use, antenatal care

## Introduction

Access to reproductive health care is a fundamental human right, recognized by leading international health organizations including the United Nations Population Fund (UNFPA) and World Health Organization (WHO) as a critical requirement for achieving universal access to health, gender equality, and reproductive equity and wellbeing (1, 2). Despite this, millions of individuals that are immigrants and those living in conflict-affected areas face systemic barriers that restrict their access to essential reproductive health care. Socioeconomic, legal, and geopolitical barriers prevent access to crucial family planning, maternal health, including abortion care services for these marginalized populations, which in turn worsen health outcomes (3–5). Furthermore, the United Nations High Commission for Refugees (UNHCR) has noted that conflict-related displacement disproportionately affects women, leaving them vulnerable to inadequate maternal health care, increased rates of unintended pregnancies, and increased exposure to gender-based violence (6). Recent research in this Research Topic underscores these disparities and emphasizes urgent policy solutions to bridge gaps in access to reproductive health care for the world's most vulnerable immigrants and individuals forcefully displaced by conflict and humanitarian situations.

## Overview of contributions

We issued a call for papers in September 2024 and received 10 submissions to this Research Topic on “*Access and Barriers to Reproductive Health Services among Immigrants and Populations in Conflict Zones*.” Following rigorous peer review, we accepted four papers for publication. In this editorial, we present an illustrative synthesis of the four peer-reviewed articles in this Research Topic. These articles include studies from Burkina Faso, Iran, and Uganda, and a systematic review which synthesizes 38 studies conducted in 20 countries, some of which are conflict-affected or destination countries for immigrants and refugees. The studies employed different research methods, including quantitative, population-based studies, policy evaluations, and mixed methods evidence synthesis. Collectively, the studies provide nuanced understanding of access and barriers to reproductive health services among immigrants and populations in conflict-affected areas.

Kawuma et al. examine gendered differences in accessing and using sexual and reproductive health services among young migrants aged 14–24 years in Southwestern Uganda. The study evaluates the “*Lending a Hand”* intervention, which trained peer supporters to provide referral advice and support, ultimately increasing sexual and reproductive health (SRH) knowledge and service utilization among young migrants. The study highlights how social, economic, and psychological challenges influence SRH risk behaviors, leading to unintended pregnancies, sexually transmitted infections (STIs), and poor mental health outcomes. Women may engage in transactional sex due to financial instability, while men may participate in risky behaviors using their earnings. Despite efforts to seek SRH services, long wait times, shortages of medicines and supplies, and financial constraints limit access to services in public and private health care facilities. The authors emphasize the importance of tailored interventions that address gendered risks and promote equitable access to reproductive health care for migrant populations (Kawuma et al.).

Munnangi et al. present an updated mixed-methods systematic review examining disrespect and abuse in health facility-based abortion and postabortion care (PAC). The evidence synthesis, based on 38 studies in 20 countries, identifies various forms of mistreatment, including stigma, discrimination, verbal and physical abuse, breaches of privacy, misinformation, and failure to meet care standards. The most frequently reported issue, inadequate or inaccurate information provided to women before, during, and after abortion procedures, compromise quality of care and patient autonomy. Additionally, the presence of protestors outside abortion facilities contribute to psychological distress and bar to access to care. The findings of this review highlight the urgent need for multi-level interventions, including healthcare provider training, policy reforms, and institutional accountability, to ensure respectful and dignified abortion care (Munnangi et al.).

Nejhaddadgar et al. examine the impact of new population policies on women's reproductive health access patterns in Ardabil, Iran, following a ban on family planning services. This comparative cross-sectional study surveyed 400 women before and 400 women after the policy change using a stratified cluster sampling method. Findings reveal a significant decline in modern contraceptive use (from 67.25% to 31%) and a rise in unwanted pregnancies (from 28.5% to 49.25%) and unsafe abortions (from 14.25% to 21.75%). The study underscores the adverse consequences of restrictive reproductive health policies, highlighting the crucial need to mitigate these effects and ensure access to safe family planning services, in line with human rights and reproductive justice (Nejhaddadgar et al.).

Badolo et al., in their study, assess trends in the utilization of antenatal care (ANC) among women of childbearing age in Burkina Faso, using data from the 2010 and 2021 Demographic and Health Surveys (DHS). Their findings reveal a significant increase in adequate ANC use, rising from 22.92% in 2010 to 46.34% in 2021, yet still falling short of WHO recommendations (7, 8). The study identifies key individual, household, and community-level factors influencing ANC access, including age, education, marital status, occupation, contraceptive use, socioeconomic status, media exposure, underlying regional disparities in ANC outcomes. Despite improvements, barriers such as healthcare infrastructure limitations and socioeconomic inequalities persist, highlighting the need for strengthening the health system to enable the provision of targeted interventions for equitable access to maternal healthcare services (Badolo et al.).

## Quality appraisal

Kawuma et al. provide rich, gender-sensitive qualitative insights into sexual and reproductive health among young migrants in two Ugandan towns through 20 in-depth interviews. However, their purposive sampling frame, recruitment via peer networks, may introduce selection bias. Moreover, loss of participants to follow-up interviews is noted by the authors as important considerations for broader generalizability of their findings (Kawuma et al.). Munnangi et al., in their mixed-methods systematic review spanning 38 studies across 20 countries, employ qualitative and quantitative appraisal tools to strengthen the thematic rigor of their review, but heterogeneity across studies in definitions of disrespect and abuse, variable measurement instruments, and exclusion of non-English or unpublished sources were described as limitations, which could potentially introduce interpretive bias. Furthermore, due to the relatively small number of quantitative studies meeting inclusion criteria, it was not feasible to do a meta-analysis (Munnangi et al.). Nejhaddadgar et al. enroll a sample of 800 married women in Ardabil to detect pre- and post-policy shifts in contraceptive use, offering strong statistical power. However, their convenience sampling at health centers cross-sectional study design, and use of an unvalidated questionnaire preclude attribution of causation and underrepresent non-clinic users (Nejhaddadgar et al.). Finally, Badolo et al. draw on nationally representative DHS data from 2010 to 2021 to investigate antenatal care trends, enhancing temporal and population coverage measurement; however, the cross-sectional design, potential five-year recall bias limit causal inference and may under- or over-estimate coverage estimates (Badolo et al.).

## Overarching themes and synthesis

Across the studies and contexts represented in this research topic, three themes are identified.

1. Gendered and intersectional barriers shape reproductive health risks and uptake of services (Kawuma et al., Munnangi et al.). Young immigrants and individuals seeking abortion services face stigma from providers attitudes and community members, which compounds socioeconomic vulnerability (3).
2. Policy shifts have a profound impact on family planning and maternal health care access and uptake (Badolo et al., Nejhaddadgar et al.). On the one hand, restrictive policies limit access to critical services and drive unsafe abortions and short-interval pregnancies in populations that are already vulnerable on many fronts. On the other hand, progressive policy shifts, for example, ANC access expansion, yield gains in health outcomes.
3. The necessity of rights-based multi-level interventions cannot be overemphasized. Ensuring quality services at the individual level must go hand-in-hand with ongoing provider training, health system accountability, and community engagement (1, Munnangi et al.). Furthermore, using data sources from different humanitarian contexts highlights that fragility in the policy ecosystem, ranging from supply chains to legal frameworks, undermines reproductive autonomy and rights for immigrants and displaced populations (9, Munnangi et al.).

## Critical gaps and future directions

Reproductive health care access should never be determined by geopolitical instability or immigration status, yet restrictive reproductive health and immigration policies, and conflict-driven health system fragility continue to marginalize already vulnerable populations. Research underscores the urgency of implementing inclusive, rights-based solutions, ensuring that immigrants and individuals in conflict-affected areas receive the care they deserve. By advancing policy, community empowerment, healthcare reform, and trauma-informed care, sustainable pathways can be created to ensure reproductive justice for all (10–12). Specifically, strengthening community-based healthcare initiatives, including telemedicine, increasing funding for reproductive health services for migrant and displaced populations, and revising restrictive reproductive health policies, are essential steps toward addressing these reproductive health inequities. Furthermore, a rights-based approach that prioritizes reproductive health care for immigrants and populations affected by conflict will not only improve reproductive health outcomes but also contribute to broader global goals, health equity, and gender equality efforts (2, 13).

In advancing access to reproductive health services for immigrants and populations affected by conflict, it is crucial to acknowledge real-world feasibility barriers. Internet connectivity gaps in camps housing displaced populations and urban settings can disrupt virtual consultations, while concerns over data privacy may discourage individuals from seeking care without robust confidentiality protections. Additionally, political resistance or shifting priorities at local and national levels can delay or hinder reforms and funding allocations. Mitigating these challenges will demand targeted infrastructure investments, culturally sensitive privacy protocols, and coordinated advocacy to build political will, ensuring that recommendations translate into accessible, secure, and sustainable services.

## Conclusion

This editorial is intended as an illustrative roadmap to key themes in access and barriers to reproductive health services among immigrants and populations in conflict-affected areas. The Research Topic highlights the complex and intersectional social, policy, and health-systems factors that drive reproductive health inequities among immigrants and populations affected by conflict. Gendered risks, policy shifts, and deficits in quality of care, recur across different study contexts. These factors underscore the need for holistic and rights-based approaches to addressing the reproductive health needs of these vulnerable populations (6, 9, 11, 14). Culturally competent healthcare providers, and telemedicine services can effectively expand reproductive health access while mitigating bureaucratic obstacles (15, 16). Strengthening community partnerships and embedding reproductive justice in provider training are key strategies for bridging service gaps. As global displacement continues, urgent investment in inclusive reproductive health research, practice, and policy would be crucial for upholding the human rights of all, regardless of immigration status or geopolitical instability.
